# Proving Nanoscale Chiral Interactions of Cyclodextrins and Propranolol Enantiomers by Means of SERS Measurements Performed on a Solid Plasmonic Substrate

**DOI:** 10.3390/pharmaceutics13101594

**Published:** 2021-10-01

**Authors:** Gabriela Fabiola Știufiuc, Valentin Toma, Anca Onaciu, Vasile Chiș, Constantin Mihai Lucaciu, Rareș Ionuț Știufiuc

**Affiliations:** 1 Faculty of Physics, “Babeș-Bolyai” University, M. Kogălniceanu 1, 400084 Cluj-Napoca, Romania; gabriela.stiufiuc@ubbcluj.ro (G.F.Ș.); vasile.chis@ubbcluj.ro (V.C.); 2 MedFuture Research Center for Advance Medicine, “Iuliu Hațieganu” University of Medicine and Pharmacy, L. Pasteur 4-6, 400349 Cluj-Napoca, Romania; valentin.toma@umfcluj.ro (V.T.); anca.onaciu@umfcluj.ro (A.O.); 3 Department of Pharmaceutical Physics-Biophysics, “Iuliu Hațieganu” University of Medicine and Pharmacy, L. Pasteur 6, 400349 Cluj-Napoca, Romania; clucaciu@umfcluj.ro

**Keywords:** cyclodextrins, chiral interactions, Raman, SERS, quantum calculations

## Abstract

Chiral separation is an important issue for the pharmaceutical industry. Over the years, several separation methods have been developed, mainly based on chromatography. Their working principle is based on the formation of transient diastereoisomers, but the very subtle nanoscale interactions responsible for separation are not always understood. Recently, Raman and surface-enhanced Raman (SERS) spectroscopy have provided promising results in this field. Here we present Raman/SERS experimental data that provide useful information concerning the nanoscale interactions between propranolol enantiomers and α, β, and γ cyclodextrins. Raman spectroscopy was used to prove the formation of host–guest intermolecular complexes having different geometries of interaction. The occurrence of new vibrational bands and a change in the intensities of others are direct proofs of complexes’ formation. These observations were confirmed by DFT calculations. By performing SERS measurements on a new type of plasmonic substrate, we were able to prove the intermolecular interactions responsible for PRNL discrimination. It turned out that the interaction strength between the substrate and the intermolecular complexes is of paramount importance for SERS-based chiral discrimination. This approach could represent a very good starting point for the evaluation of molecular interactions manifesting between other pharmaceutical compounds and different classes of chiral selectors.

## 1. Introduction

Chiral discrimination is a very intriguing characteristic of living systems that could hold the key for a precise understanding of living matter. More specifically, the ability to generate concluding scientific proof of the possible mechanism involved in chiral discrimination needs to be properly addressed, especially from an experimental point of view. Over the years, this research topic has drawn immense attention in the pharmaceutical sciences. The pharmaceutical industry is highly interested in finding new experimental techniques able to discriminate and, ideally, separate the naturally occurring pharmaceutical enantiomers. Among the different techniques that have been intensively applied in analytical separation of pharmaceutical compounds, the chromatographic ones are by far the most utilized. The fundamental process responsible for chiral separation and discrimination of pharmaceutical enantiomers is the formation of a transient diastereomeric complex between the pharmaceutical enantiomers and a chiral selector. The main physical interactions involved in the formation of such a complex are hydrogen bonds; ionic, ion–dipole or dipole–dipole; and van der Waals or π–π interactions [1].

The scientific literature is abundant in papers dealing with the analytical applications of different enantioseparations techniques [2,3], but the use of Raman and its counterpart, surface-enhanced Raman spectroscopy (SERS), towards chiral discrimination has not been often reported. Raman spectroscopy is a particularly sensitive technique that has proved its capabilities in different areas of pharmaceutical applications [4,5] based on its unique ability to provide vibrational fingerprint of the pharmaceutical compounds. It entails minimal sample preparation; very small amounts of analytes and the nondestructive measurements can be performed in aqueous solutions. The development of new synthesis methods for the fabrication of different plasmonic nanostructures led to a massive increase in the vibrational molecular signal collected in a Surface Enhanced Raman Spectroscopy (SERS) experiment. Several studies have shown that SERS is capable to identify molecular species in ultra-low concentration: the final goal being single molecule detection in solutions. In a paper published in 2006, Le Ru et al. [6] unequivocally showed that SERS possesses single-molecule sensitivity for measurements performed in solution on a standard analyte. However, every magical story has a drawback that hampers its implementation in large scale application and up to now, in the case of pharmaceutical applications of Raman/SERS, this major drawback is the lack of reproducibility of the recorded spectra. 

In addition, the scientific literature has plenty of papers reporting the synthesis of different plasmonic substrates [7,8,9], without any consensus on how the reproducibility problem can be overcome. The problem intensifies for SERS-based chiral selectivity applications, and a very scarce number of publications can be found for this particular application [10,11]. Over time, our group performed intensive research activities, trying to elucidate this crucial aspect of paramount importance for pharmaceutical industry. In a paper published in 2015, we demonstrated for the first time that SERS can be implemented for studying the chiral interactions between propranolol (PRNL) enantiomers and the three classes of native cyclodextrins (CDs) by using colloidal silver and gold nanoparticles as plasmonic substrates [12]. The main conclusion of that study was that the nature of the colloidal substrate employed in the recording of the experimental spectra has a major role in SERS-based chiral discrimination applications. 

Most recently, we developed a new type of solid plasmonic substrate that was employed for recording highly reproducible SER spectra of blood samples. These spectra were further used for early cancer detection, using a SERS-based method combined with multivariate analysis of the enhanced vibrational spectra collected on this very complex analyte: blood plasma. We showed that our consistently reproducible spectra generated by the substrates allowed the discrimination between breast cancer patients and controls, with a sensitivity of 90%, a specificity of 89%, and an accuracy of 89% [13]. These excellent values of the three parameters are mainly due to the quality of the substrates used in the experiment. 

In the present study, we performed SERS measurements on the complexes formed by the two enantiomers of propranolol (R/S-PRNL) with αCDs, βCDs, and γCDs, using this new type of plasmonic substrate. The experimental spectra and the quantum chemistry calculations allowed the nanoscale evaluation of the specific interactions of the host–guest intermolecular complexes, both qualitatively and semi-quantitatively. A clear identification of the molecular mechanism responsible for the βCD-mediated chiral discrimination between PRNL’s S and R enantiomers is reported. We consider that a proper understanding of the intermolecular interactions responsible for chiral discrimination is the first condition that needs to be fulfilled in order to develop new, rapid and simple techniques for this type of application. In principle, the SERS-based approach presented in this study could be used in the case of any complexes formed between a pharmaceutical compound and different chiral selectors.

## 2. Materials and Methods

### 2.1. Materials 

All the chemicals employed in this study were of analytical grade. During the synthesis process they were used without any further purification. Silver nitrate (AgNO_3_) and hydroxylamine (NH_2_OH) were purchased from Roth GmbH (Karlsruhe, Germany). The aqueous solutions were prepared by using ultrapure water (18.2 MΩ × cm, Chorus PureLabElga, Lane End, High Wycombe, UK). CaF_2_ polished glasses (20 mm diameter and 1 mm thickness) were purchased from Crystran Limited, Poole, UK. They were employed as port-probes for the synthesis of the solid plasmonic substrates.

### 2.2. Synthetic Procedures

#### 2.2.1. Hydroxylamine-Reduced Silver Colloid Synthesis 

Hydroxylamine-reduced silver colloids (AgHyam) were synthesized at room temperature, according to a procedure slightly modified from the one proposed by Leopold and Lendl [14]. Briefly, 10.5 mg of hydroxylamine hydrochloride and 12 mg of sodium hydroxide were dissolved in 90 mL of ultra-purified water. In a second bottle, 17 mg of silver nitrate was dissolved in 10 mL of ultra-purified water, and the obtained solution was quickly poured into the previous one, under vigorous stirring. The color of the final solution quickly changed from colorless to brown and finally to yellowish gray in the course of 5 min. Once the synthesis process was completed, the nanoparticles were purified and concentrated by using the tangential flow filtration (TFF) technique, according to a procedure described in the literature [15,16]. After the TFF purification step, the AgHyam NPs were used directly for the synthesis of the solid plasmonic substrates. 

#### 2.2.2. Synthesis of the Plasmonic Solid Substrates

The TFF-processed colloidal solutions were used for the synthesis of solid plasmonic substrates created on top of CaF_2_ glasses. Prior to NPs deposition the CaF_2_ glass were prepared by cleansing with acetone and ethanol, rinsing with ultrapure water and left to air dry. After 15 min, they were heated at 40 °C, using a plate heater. In the final step, a 1 µL volume of concentrated colloid was pipetted on the CaF_2_ port-probe and left to dry for several minutes. The as-obtained SERS solid substrates, having a mean diameter of ~1 mm, were removed from the heated plate and cooled down to room temperature.

### 2.3. Methods

The nanoparticles, solid plasmonic substrates, propranolol enantiomers, and intermolecular complexes were characterized by UV–Vis absorption spectroscopy, TEM imaging, Photon Correlation Spectroscopy (PCS), Nanoparticle Tracking Analysis (NTA), Raman and Surface Enhanced Raman spectroscopy measurements.

Electron microscopy measurements were performed on a HT7700 (Hitachi, Tokyo, Japan) Transmission Electron Microscope (TEM) operating at 100 kV, using the high-resolution operation mode (spot size 3 = 0.60 μm). Samples were deposited on carbon films on top of copper grids for 2–5 min depending on sample type and concentration. After deposition, the excess solution was blotted away, using No. 42 ash-less filter paper, and the grids were left covered at room temperature to completely dry. The obtained images were calibrated for size, annotated, and processed (contrast enhancement by histogram stretching, smoothing (sigma = 2.2), and cropping) in ImageJ2 v1.53 [17].

The Photon Correlation Spectroscopy (PCS) analysis was performed on a Vasco^γ^ nanoparticle analyzer (Cordouan Technologies, Pessac, France), using a monochromatic laser beam with a wavelength of 658 nm and 65 mW power. 

Nanoparticle Tracking Analysis (NTA) measurements were performed by using a ZetaView^®^ NTA-Nanoparticle Tracking Video Microscope PMX-120 (Particle Metrix GmbH, Inning am Ammersee, Germany). A 1000-times dilution was performed for all colloids with ultra-purified water, before injecting the samples in the device. The recording parameters were automatically set before each analysis.

The Raman/SERS measurements were recorded by using a confocal Renishaw^®^inVia microscope (Renishaw plc, Wotton-under-Edge, Gloucestershire, UK), equipped with a Leica microscope (Leica Microsystems GmbH, Wetzlar, Germany), using a 50× objective (N.A. 0.75). A 785 nm diode laser (Renishaw, Wotton-under-Edge, UK) was used for excitation. Prior to each set of measurements a calibration procedure was performed by using an internal silicon reference. The laser power (measured at the sample surface) was ~113 mW for Raman and 1.07 mW (measured at the sample surface) for SERS measurements. The acquisition time was set to 40 s (10 s integration time and 4 accumulations) for both types of measurements. The spectrograph was equipped with a 600 lines/mm grating and a charge coupled device camera (CCD). The spectral resolution of the spectrometer was ~2 cm^−1^. Each spectrum represents the average of minimum 30 spectral acquisitions, collected on different randomly chosen regions of the sample. The baseline correction was performed by using the Wire 4.2 software provided by Renishaw plc (Wotton-under-Edge, Gluocestershire, UK), with the inVia spectrometer. The results were processed by using OriginPro 2019 software (OriginLab, Nothampton, Massachusetts, USA).

R/S-PRNL and CDs stock solutions used for Raman/SERS measurements were prepared to 1 mM concentration and were mixed at different volume ratios (0–100%). For Raman measurements, we used 1 µL of the as-prepared solutions; it was poured and dried on a clean aluminum foil stuck on a glass slide. In the case of SERS measurements, 1 µL of sample was poured and dried on our solid plasmonic substrates.

All the calculations related to molecular geometry optimization and generation of theoretical vibrational spectra were performed by using the Gaussian 09 software package [18] based on density functional theory (DFT) methods at the B3LYP/6-311+G (d, p) level of theory [19,20]. No symmetry restrictions were applied during the optimization process. The calculated Raman activities (*S_i_*) were converted into relative Raman intensities (*I_i_*), using the relation given below [21,22]:

(1) $$I_{i}=\left[f{\left(\upsilon_{0}-\upsilon_{i}\right)}^{4}S_{i}\right]/\left\{\upsilon_{i}\left[1-exp\left(-hc\upsilon_{i}/k_{\beta }T\right)\right]\right\}$$

where *f* is a normalization factor for all peak intensities, *υ_0_* represents the exciting laser wavenumber, *υ_i_* is the wavenumber of the *i*th vibrational mode, *c* stands for the speed of light, *h* and *k_β_* are Planck’s and Boltzmann’s constants, and *T* represents the temperature.

Since the vibrational wave numbers obtained by quantum chemical calculation are typically larger than their experimental counterparts, the computed wave numbers for the frequencies larger than 1000 cm^−1^ were scaled by 0.967, as proposed by Scott and Radom [23].

## 3. Results and Discussion

The formation of the complexes was obtained by mixing equal volumes of desired enantiomers and CDs aqueous solutions, having the same concentration (1 mM). The mixtures were kept at room temperature for several hours in order to allow the formation of 1:1 host–guest intermolecular complexes, according to a procedure that was previously reported [12]. This observation was confirmed by NMR measurements [24].

The solid plasmonic substrates were prepared in our laboratory, using as building blocks hydroxyl amine reduced silver nanoparticles (AgNPs) filtered and concentrated by means of TFF, according to a procedure described in detail in the literature [13]. A typical TEM image of the purified nanoparticles is presented in Supplementary Materials Figure S1. The statistical analysis, performed on a large number of NPs from the TEM images, indicated a mean diameter value of ~33 nm and a high degree of mono-dispersity (inset of Supplementary Materials Figure S1). This value was also confirmed by PCS measurements. After the TFF purification step the concentration of the AgNPs was ~ 4 × 10^10^ NPs/mL, as indicated by NTA. Nevertheless, the TFF purification improved the dimensional polydispersity of AgNPs, as it was observed from the UV–Vis absorption measurements recorded before and after colloidal purification [13]. By removing the chemical by-products generated during the synthesis process, the interaction of the molecular complexes with the plasmonic substrate becomes more “predictive” and allows us to record very reproducible SER spectra. 

**Figure 1:**
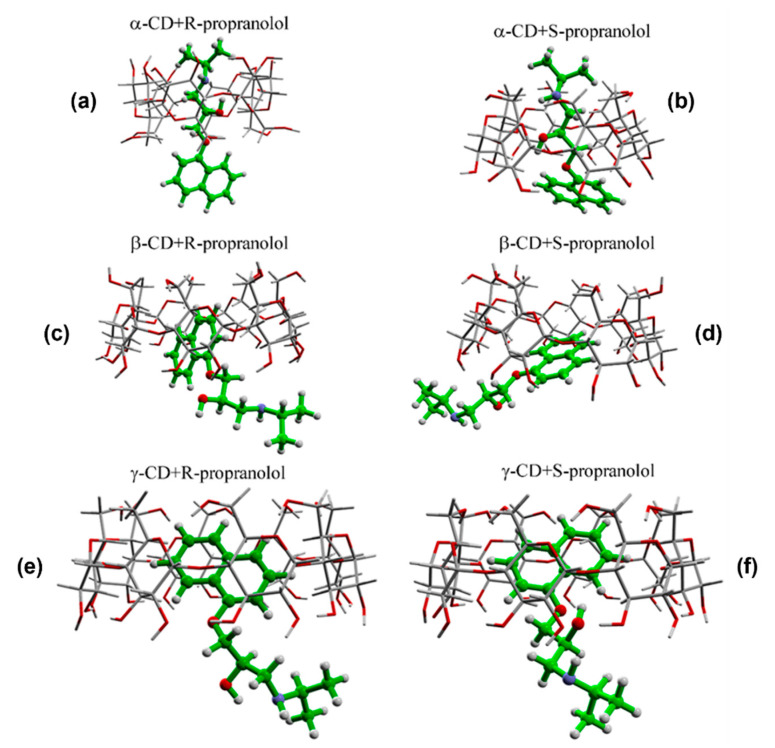
Calculated geometries of interactions of the six intermolecular complexes formed between PRNL and cyclodextrins: α-CD+R-propanolol (**a**), α-CD+S-propanolol (**b**), β-CD+R-propanolol (**c**), β-CD+S-propanolol (**d**), γ-CD+R-propanolol (**e**), γ-CD+S-propanolol (**f**).

Quantum chemical calculations performed at the B3LYP-D/6-31G (d) level of theory showed that, from a theoretical point of view, the interaction between PRNL enantiomers and α, β, and γ CDs will lead to the formation of six 1:1 host–guest intermolecular complexes having different geometries of interaction. In the case of αCDs complexes, they were produced as a result of the incorporation of the alkyl chain into the hydrophobic cavity of CDs, whereas, in the case of β and γ CDs, the naphthalene moiety were more or less incorporated into the hydrophobic cavity. The calculated geometries of the six classes of intermolecular complexes are shown in Figure 1.

As it can be seen in the figure, the interaction between R/S-PRNL and αCD and γCD are very similar for both enantiomers. In the case of R/S-PRNL/αCDs complexes, the PRNL incorporation takes place through the alkyl chain, whereas, in the case of R/S-PRNL/γCDs complexes, the naphthalene moiety is included inside the cavity. This behavior is a direct consequence of rim’s diameter values (~5 Å in the case of αCDs vs. ~8 Å for γCDs) highlighting the major role played by the spatial effects in chiral discrimination. In a recent paper, Banerjee-Ghosh et al. suggested that chiral recognition and discrimination in nature and/or artificial systems depends solely on spatial effects [25]. In our case, this assumption is valid in the case of the complexes formed with βCD. Even though the calculated energies of interaction between R/S enantiomers and βCDs are almost identical (−36.2 kcal/mole for R–βCD vs. −32.8 kcal/mole for S–βCD complex), the interaction geometry is quite different. More precisely, in the case of the R–βCD complex the naphthalene ring is “fully” incorporated into the cavity, whereas, for the S–βCD complex, it is only partially incorporated (Figure 1c,d). This very subtle difference in the interaction mechanism of PRNL enantiomers and βCDs was experimentally proven by Raman/SER spectroscopy, as is explained in the next paragraphs, highlighting the enormous potential of SERS to experimentally prove molecular nanoscale interactions that could have a great impact on the understanding the mechanism responsible for chiral separation.

### 3.1. Raman Analysis

For a proper experimental assignment of these differences, we performed Raman spectroscopy measurements on pure enantiomers and on each type of complex they form with CDs. The measurements were carried out in the same experimental conditions. Prior to any measurements, the aqueous solutions having the same concentration of analytes were poured onto a CaF_2_ substrate and allowed to dry. We observed that the intensity of the vibrational peaks varies linearly with the laser power, so the vibrational intensities were plotted in kcts/(mW × s) units. This representation allows a quantitative insight of the intermolecular interactions between PRNL enantiomers and CDs. 

**Figure 2:**
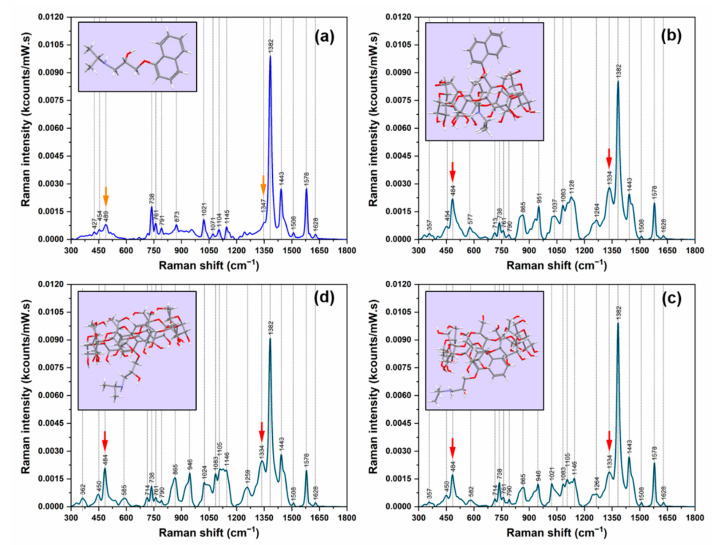
Raman spectra of R-PRNL (**a**) and of the complexes it forms with αCD (**b**), βCD, (**c**) and γCD (**d**). The spectra were recorded by using an excitation laser of 785 nm. The arrows indicate the two bands specific for the complexes. The insets show the calculated geometries of inclusions.

The Raman spectra of pure R-PRNL and of the complexes it forms with αCDs, βCDs, and γCDs are presented in Figure 2. In the Supplementary Materials section, we plotted the same spectra for the S-enantiomer (Supplementary Materials Figure S2), and the results are quite similar. As it has been previously reported [26], the Raman spectrum of R-PRNL pure enantiomer is dominated by the vibrational bands assigned to naphthalene ring vibrations: 1382 cm^−1^ (δ_in-plane_), 1578 cm^−1^ (ν_C=C_), 1443 cm^−1^ (ν_C–C_), and 738 cm^−1^ (naphthalene breathing mode). Upon the formation of the three molecular complexes, one can detect an immediate modification of their Raman spectra. They are very similar in the case of the three complexes but different with respect to the Raman spectrum of pure enantiomer. The first observation is the increase of the vibrational band assigned to an in-plane vibration (489/484 cm^−1^) and the decrease of the 738 cm^−1^ breathing mode, both being assigned to naphthalene ring. The most intense vibrational peak (1382 cm^−1^) is the same for all the spectra.

The shift observed for the in-plane vibration mode (489 cm^−1^ in the case of pure enantiomers vs. 484 cm^−1^ in the case of the complexes) may be due to conformational changes induced by the inclusion process, as it was previously shown in the case of the other molecules that form complexes with CDs [27]. According to our DFT calculations, this peak was assigned to symmetric longitudinal stretching vibrations of the naphthalene ring. This represents the first experimental proof of the successful formation of the three classes of complexes. 

From the point of view of its intensity, this peak presents also a very interesting behavior. In the case of pure enantiomers, it is barely visible, whereas, for the complexes, it is strongly enhanced. As it can be seen in the figure, its intensity increases almost 300% (from ~0.07 kcts/(mW × s) to ~0.20 kcts/(mW × s)) upon the formation of the complexes. 

The most evident spectral changes are detected in the most intense spectral region 1300−1500 cm^−1^. As it can be seen in Figure 2, the 1334 cm^−1^ vibrational peak is the most meaningful one, being another direct piece of evidence of the formation of the intermolecular complexes. This peak can be assigned to a bending vibration of the CH group of the methylamino chain coupled to a waging vibration of the CH_2_ group [26]. In the theoretical and experimental Raman spectra of pure PRNL enantiomers, it appears as a barely distinguishable shoulder of the most dominant vibrational band (the naphthyl ring vibration mode @ 1382 cm^−1^) (Figure 2a). Upon the formation of the complexes, it becomes a distinct peak in the Raman spectra of R/S-PRNL/αCD, R/S-PRNL/βCD, and R/S-PRNL/γCD complexes. A similar shift with the one observed for the in-plane vibration mode of the naphthalene ring (489/484 cm^−1^) is also detected in this case (1347 cm^−1^ for pure enantiomer vs. 1334 cm^−1^ for the complexes). 

The Quantum Chemical calculations indicated that, in the case of the complexes formed with αCDs, the alkyl chain will be incorporated in the hydrophobic cavity. This type of interaction, valid for both enantiomers, can be probably attributed to the very small internal diameter of αCD (~5 Å). In this situation, the naphthalene ring will be “left” outside the cavity. A direct consequence of this geometry of inclusion is the decrease of the intensity of the most intense vibrational band (1382 cm^−1^) associated to a naphthalene ring vibrational mode. This decrease is accompanied by a strong increase of the 1334 cm^−1^ band which becomes the second most intense vibrational band of the complexes formed with αCDs. Based on similar reports for Cucurbituril (CU) molecules, where a vibrational band (belonging to the cavity) can be used as a marker for determining CU molecular loading [28], we believe that the intensity of the 1382 cm^−1^ band can provide useful information related to PRNL inclusion in CDs.

As a first conclusion, one can say that, in this study, Raman spectroscopy was successfully used for proving the formation of the 1:1 host–guest intermolecular complexes between PRNL and CDs. The two vibrational bands that presented a strong increase of their intensity and a shift of their position (marked with an arrow in Figure 2), assigned to naphthalene ring and methylamino chain vibrations, respectively, are a direct experimental evidence of this process.

### 3.2. SERS Analysis

PRNL molecule has a chiral center located right next to the naphthalene ring. From a spectroscopic point of view, the molecule can be considered as being composed of two major components: the aromatic naphthyl ring and the alkyl chain. In a previous paper, Farcas et al. [26] pointed out the major role played by the aromatic ring in the vibrational spectra collected on four classes of β-blockers having a very similar structure: atenolol, metoprolol, bisoprolol, and propranolol. It was also shown that Raman/SERS spectroscopy is very sensitive with respect to the type of the central ring of the molecule. As such, the replacement of the phenyl ring with naphthalene produced a dramatic change in the molecular vibrational pattern [26].

**Figure 3:**
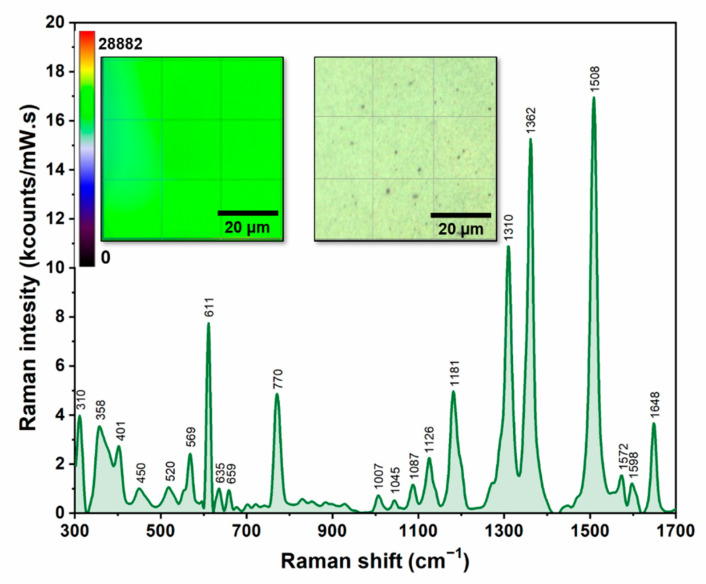
SER spectrum of R6G analyte recorded by using a 785 nm laser. The left inset shows the heat map created by using the intensity of the 1508 cm^−1^ band. The right inset presents a 60 × 60 μm^2^ optical image of the substrate.

In this study we tried to understand how a very subtle difference between the interactions of the PRNL enantiomers (having the same chemical structure) with the three classes of CDs can be explained by means of Raman/SERS. The novelty of this study relies on the fact that SERS measurements were performed on dried samples that were previously poured on a new type of solid plasmonic substrate that was fabricated by using TFF purified silver nanoparticles, according to a procedure described in detail in the literature [13]. The reproducibility capacities of the substrate were firstly tested on a “typical” Raman analyte: rhodamine 6G (R6G). In this case, the spectra were collected on a 60 × 60 μm^2^ area of the substrate, using an excitation laser of 785 nm, in the same experimental conditions. As it can be seen in Figure 3, the variation of the most intense peak of R6G (1508 cm^−1^) was less than 10%. The heat map, created by using these values, is presented in the inset of Figure 3, together with an optical image of the substrate.

**Figure 4:**
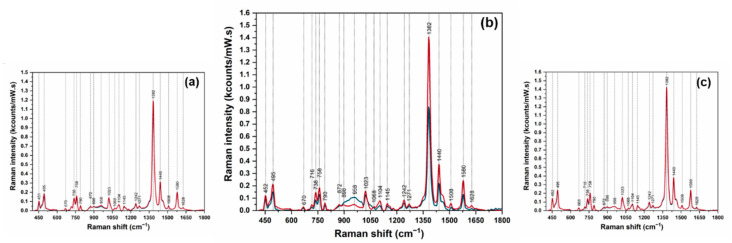
SER spectra of the host–guest complexes formed between R/S-PRNL and αCDs (**a**), βCDs, (**b**) and γCDs (**c**). The complexes formed with R-PRNL and S-PRNL are plotted in blue and red, respectively.

The solid plasmonic substrates were further used for collecting the SER spectra of the pure enantiomers (Supplementary Materials Figure S3) and of the complexes with CDs. The SER spectra of the six classes of intermolecular complexes PRNL/CDs are plotted in Figure 4.

The high quality of the SER spectra is the first observation that can be made at a careful examination of all spectra. It is a direct consequence of the solid plasmonic substrates employed in this study. The measurements were performed on dried analytes, so one can assume that the intermolecular complexes are located in the very close vicinity of the plasmonic substrate. In the case of the complexes formed with αCDs (Figure 4a), the two spectra are identical from the point of view of the shape, as well as band intensities. This is a strong evidence that the interaction geometries of PRNL enantiomers and αCDs molecules are identical. The most intense vibrational band remains the one located at 1382 cm^−1^. This band was also the most intense one in the Raman spectrum of the complexes. This is a clear evidence that the intermolecular complexes do not chemisorb on the surface, since we do not see any band shift. Based on the surface selection rules developed by Moskovits [29], one can believe that the evaporation of water molecules brought the complexes closer to the plasmonic surface but the formation of any chemical bonds between them is highly improbable. A similar behavior was observed in the case of R/S-PRNL, when using as substrates colloidal nanoparticles [4]. Thus, the only enhancement mechanism that can be responsible for the amplification of the vibrational bands is the electromagnetic one [29].

The formation of R/S-PRNL/γCDs complexes implies the inclusion of the naphthalene moiety into CD cavity, for both enantiomers. As expected, the SER spectra of the two complexes are identical (Figure 4c). This observation was also made in Raman experiments (Figure 2d). On the other hand, a direct consequence of naphthalene ring inclusion in the cavity is the increase of the most intense vibrational band (1382 cm^−1^), assigned to a naphthyl ring vibration mode. By comparing this value with the one measured in the case of αCDs complexes, when the naphthalene ring lies outside the cavity, one observes a ~15% increase (1.4 vs. 1.2 kcts/(mW × s)).

The most interesting results were observed in the case of the complexes formed with βCDs (Figure 4b). As it was pointed out before, βCDs are used for chiral discrimination of PRNL enantiomers in different electro-driven techniques [30]. The mechanism responsible for the discrimination relies mainly on different interaction geometries of PRNL enantiomers with βCD molecules. This finding is supported by the quantum chemical calculations presented in Figure 1b,c. Moreover, the SERS data presented in Figure 4 highlight that, only in the case of the PRNL/βCDs complexes, the spectral signatures of the two complexes are different. In the case of the S-PRNL/βCD complex, the spectrum is very similar to the one observed for the rest of the complexes (red spectrum in Figure 4b). The intensity of the 1382 cm^−1^ band is very close to the one observed in the case of the R-PRNL/γCD complex, indicating that water evaporation favors a similar interaction geometry. For the R-PRNL/βCD complex, the intensity of the same vibrational band is much lower (0.8 kcts/(mW × s) vs. 1.4 kcts/(mW × s)). This observation is quite unusual, since the calculated geometries of interaction suggest that in the case of R–βCD complex the naphthalene ring has a higher degree of incorporation into the CDs cavity with respect to the S–βCD complex. At a careful examination of the two spectra presented in Figure 4b one can observe that spectral differences can also be detected in the spectral interval where the vibrational modes of the alkyl chain are dominant: 850–1000 cm^−1^. The only explanation for this behavior could be that in the case of the R/S–βCDs complexes the geometry of interaction between the two enantiomers and the βCD molecule is governed by the interaction between the chain and the CD’s rim. In this case, the evaporation of water molecules can pull in or drag out the naphthalene ring from the CD’s cavity and the 1382 cm^−1^ band can be used as a very efficient “sensor” capable to monitor the degree of inclusion.

**Figure 5:**
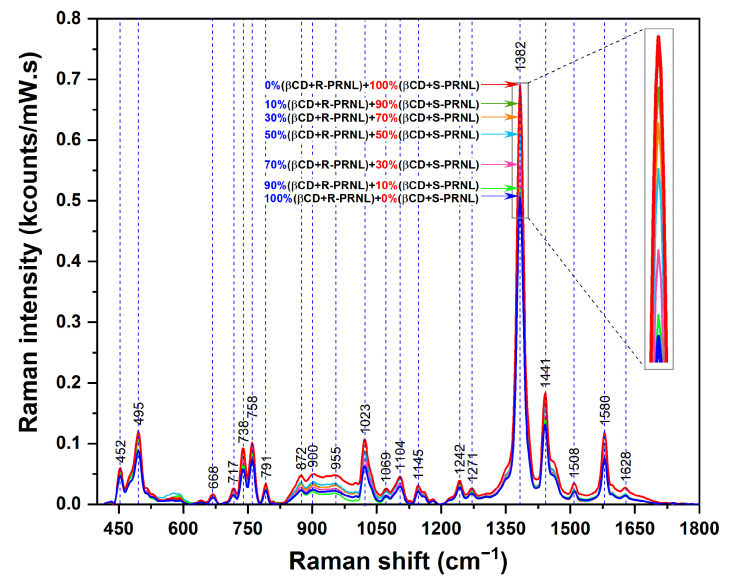
SERS spectra of pure R–βCDs (blue curve) complexes, S–βCDs (red curve) complexes and of mixed solutions containing different ratios of R/S–βCDs complexes (green, magenta, light blue, orange, and olive curves). The spectra were collected by using a 785 nm wavelength on the same plasmonic substrate. The inset highlights the intensity of the 1382 cm^−1^ band.

**Figure 6:**
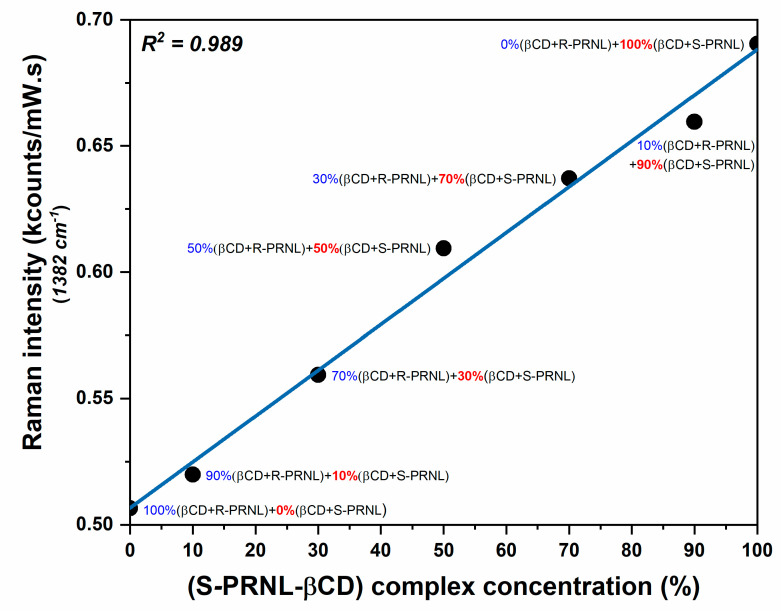
Intensity variation of the most prominent vibrational band (1382 cm^−1^) as a function of S-PRNL/βCD concentration. The intensity values were taken from the spectra presented in Figure 5.

In order to get more insights of this interesting phenomenon and to show that SERS can be used for quantitative evaluation of the presence of the two enantiomers, we performed SERS measurements on mixed solutions containing different amounts of R–βCDs and S–βCDs complexes: pure R–βCDs, 10%S–βCDs/90%R–βCDs, 30%S–βCDs/70%R–βCDs, 50%S–βCDs/50%R–βCDs, 70%S–βCDs/30%R–βCDs, 90%S–βCDs/10%R–βCDs, and pure S–βCDs. The SER spectra of the 7 solutions are presented in Figure 5. The inset highlights the intensities of the dominant vibrational band (@1382 cm^−1^). The intensities this band in the case of the mixed solutions, containing both R–βCD and S–βCD complexes in different ratios, are comprised between the intensities collected for pure S/CDs complexes (the most intense, red curve) and pure R–βCDs complexes (the less intense, blue curve). Moreover, the intensity of this mode increases by increasing the amount of S–βCDs complexes in the solutions. By plotting the intensity of 1382 cm^−1^ band as a function of S–βCDs concentration one can find a linear dependence, supporting the previous assumption. The fact that, in the case of S–βCDs complexes, the naphthalene ring has a higher degree of mobility allows its incorporation into the cavity into a more favorable conformation as a result of the complexes’ interaction with the plasmonic substrate. As such, the intensity of this mode linearly increases with the S-PRNL/βCD concentration, as can be seen in Figure 6. 

Due to a stronger interaction of R-PRNL with βCDs, the R-PRNL/βCD complex interacts slightly different with the plasmonic substrate as compared to S-PRNL/βCDs. As a result, when measuring mixed solutions containing R/S–βCDs complexes, the intensity of the 1382 cm^−1^ band has a linear dependence on S–βCD concentration, showing that this band can be used for evaluating the ratio between the two complexes. This very subtle difference was experimentally proven by means of SERS as a further proof of its enormous potential in chiral separation applications. 

## 4. Conclusions

In conclusion, in this study, we employed Raman/SER spectroscopy together with quantum chemical calculations in order to understand the nanoscale interactions between propranolol enantiomers and α, β, and γCDs. The successful formation of the intermolecular complexes was experimentally proven by means of Raman spectroscopy. The SERS experiments were carried out on a new type of solid plasmonic substrate that is capable of generating very reproducible spectra for all kinds of analytes. It was shown that, in the case of the complexes formed with αCDs and γCDs, the geometries of interaction are identical for both enantiomers. This leads to identical Raman and SER spectra for the complexes formed between R and S PRNL with αCDs and γCDs, respectively. In the case of the complexes formed with βCDs, we identified a specific vibrational band assigned to the naphthyl ring vibration of PRNL (1308 cm^−1^) that can be employed in order to quantitatively evaluate the concentration of S-PRNL/βCDs complexes present in a mixed solution containing both types of complexes. Moreover, the quantum chemical calculations showed that the weaker interaction existing between S-PRNL and CDs, as compared to R-PRNL, leads to the formation of a more “flexible” complex. The subsequent interaction of this molecular complex with the plasmonic substrate can pull in the naphthalene ring into a CD’s cavity, and the 1382 cm^−1^ band can be used as a very efficient “sensor” that is capable of monitoring the degree of inclusion and, hence, the concentration of S-PRNL/βCDs complexes.

We truly believe that this approach can pave the way for further studies concerning the understanding of nanoscale interactions between other chiral molecules and different classes of chiral selectors.

## Data Availability

Data is contained within the article or supplementary material.

## Supplementary Materials

The following are available online at https://www.mdpi.com/article/10.3390/pharmaceutics13101594/s1. Figure S1: TEM image of TFF purified AgHyam NPs. The inset shows the size distribution plot of the NPs. Figure S2: Raman spectra of S-PRNL (a) and of the complexes it forms with αCD (b), βCD (c), and γCD (d). The spectra were recorded by using an excitation laser of 785 nm. The arrows indicate the two bands specific for the complexes. The insets show the calculated geometries of inclusions. Figure S3: SERS spectra of S-PRNL (red line) and R-PRNL (blue line) recorded by using a 785 nm excitation laser.
