# Supplementary material for: Proving Nanoscale Chiral Interactions of Cyclodextrins and Propranolol Enantiomers by Means of SERS Measurements Performed on a Solid Plasmonic Substrate
Source: Pharmaceutics. 2021 Oct 1;13(10):1594. doi: 10.3390/pharmaceutics13101594 (PMC8539071; doi:10.3390/pharmaceutics13101594)
Supplement: Supplementary file 1 [file pharmaceutics-13-01594-s001.zip › pharmaceutics-1377541-supplementary.pdf]

pharmaceutics

Supplementary Materials: Proving nanoscale chiral interactions of cyclodextrins and propranolol enantiomers by means of SERS measurements performed on a solid plasmonic substrate

Gabriela Fabiola Știufiuc, Valentin Toma, Anca Onaciu, Vasile Chiș, Constantin Mihai Lucaciu and Rareș Ionuț Știufiuc

Citation: Știufiuc, G.F.; Toma, V.; Onaciu, A.; Chiș, V.; Lucaciu, C.M.; Știufiuc, R.I. Proving Nanoscale Chiral Interactions of Cyclodextrins and Propranolol Enantiomers by Means of SERS Measurements Performed on a Solid Plasmonic Substrate. *Pharmaceutics* **2021**, *13*, 1594. https://doi.org/10.3390/pharmaceutics13101594

Academic Editor: Ferenc Fenyvesi and Judit Váradi

Received: 27 August 2021
Accepted: 27 September 2021
Published: 1 October 2021

Publisher's Note: MDPI stays neutral with regard to jurisdictional claims in published maps and institutional affiliations.

Copyright: © 2021 by the authors. Licensee MDPI, Basel, Switzerland. This article is an open access article distributed under the terms and conditions of the Creative Commons Attribution (CC BY) license (http://creativecommons.org/licenses/by/4.0/).

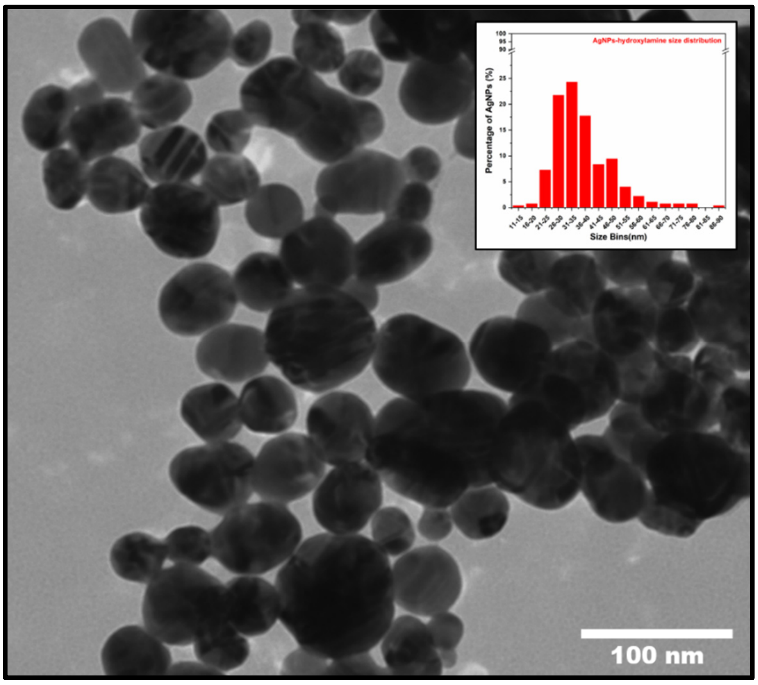

Figure S1. TEM image of TFF purified AgHya NPs. The inset shows the size distribution plot of the NPs.

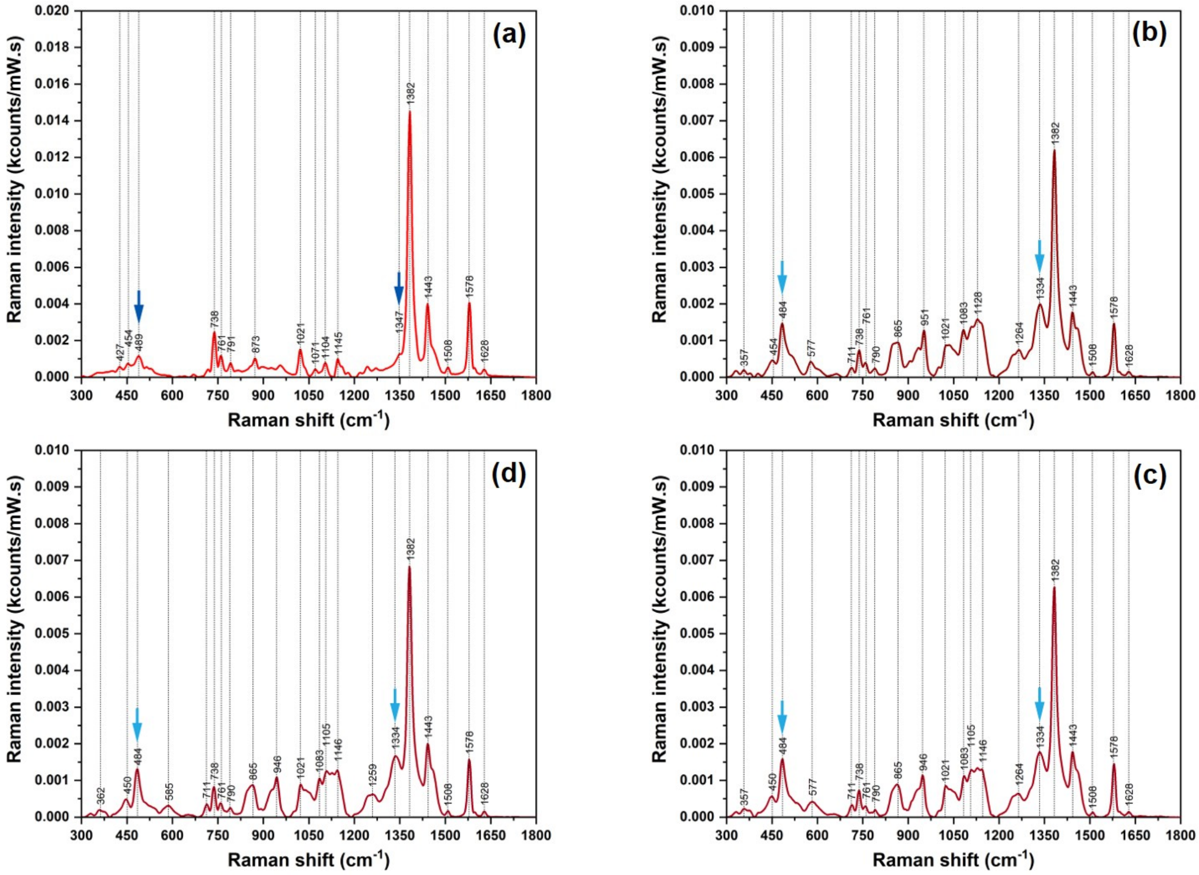

Figure S2. Raman spectra of S-PRNL (**a**) and of the complexes it forms with α-CD (**b**), β-CD (**c**) and γ-CD (**d**). The spectra have been recorded using an excitation laser of 785 nm. The arrows indicate the two bands specific for the complexes.

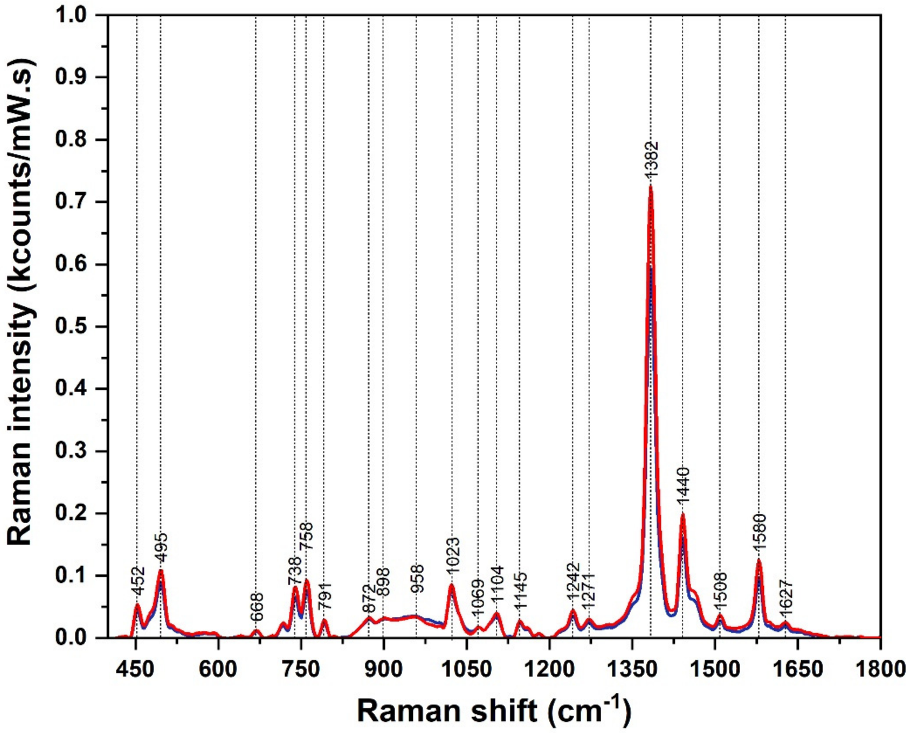

Figure S3. SERS spectra of S-PRNL (red line) and R-PRNL (blue line) recorded using a 785 nm excitation laser.
